# Improvement of *Medicago sativa* Crops Productivity by the Co-inoculation of *Sinorhizobium meliloti*–Actinobacteria Under Salt Stress

**DOI:** 10.1007/s00284-021-02394-z

**Published:** 2021-03-01

**Authors:** Samira Saidi, Hafsa Cherif-Silini, Ali Chenari Bouket, Allaoua Silini, Manal Eshelli, Lenka Luptakova, Faizah N. Alenezi, Lassaad Belbahri

**Affiliations:** 1 Laboratory of Applied Microbiology, Department of Microbiology, Faculty of Natural and Life Sciences, University Ferhat Abbas Setif, Sétif, Algeria; 2 Plant Protection Research Department, East Azarbaijan Agricultural and Natural Resources Research and Education Center, AREEO, Tabriz, Iran; 3 Food Science and Technology Department, Faculty of Agriculture, University of Tripoli, Tripoli, Libya; 4 Department of Biology and Genetics, Institute of Biology, Zoology and Radiobiology, University of Veterinary Medicine and Pharmacy, Kosice, Slovakia; 5 Department of Environmental Technology Management, College of Life Sciences, Kuwait University, Safat, Kuwait; 6 NextBiotech, 98 Rue Ali Belhouane, Agareb, Tunisia; 7 Laboratory of Soil Biology, University of Neuchatel, Neuchâtel, Switzerland

## Abstract

Biotic and abiotic stresses are severely limiting plant production and productivity. Of notable importance is salt stress that not only limits plant growth and survival, but affects the soil fertility and threatens agricultural ecosystems sustainability. The problem is exacerbated in fragile arid and semi-arid areas where high evaporation, low precipitation and the use of salty water for irrigation is accelerating soil salinization. Legumes, considered very nutritious foods for people and providing essential nutrients for ecosystems are a fundamental element of sustainable agriculture. They can restore soil health by their ability to fix nitrogen in a symbiotic interaction with the rhizobia of the soil. However, salt stress is severely limiting productivity and nitrogen fixation ability in legumes. Plant growth-promoting rhizobacteria (PGPR) and mainly actinobacteria promote plant growth by producing phytohormones, siderophores, antibiotics and antifungal compounds, solubilizing phosphate and providing antagonism to phytopathogenic microorganisms. In addition, actinobacteria have beneficial effects on nodulation and growth of legumes. In this study, actinobacteria isolated from different niches and having PGP activities were used in co-inoculation experiments with rhizobia in *Medicago sativa* plants rhizosphere submitted to salt stress. The results indicate that drought- and salinity-tolerant Actinobacteria with multiple PGP traits can potentially increase alfalfa growth under saline conditions, in the presence or absence of symbiotic rhizobial bacteria. Actinobacteria discovered in this study can, therefore, be suitable biofertilizers in the formulation of agricultural products improving plant development, health and productivity in saline soils, a necessary alternative for modern agriculture and sustainable development.

**Supplementary Information:**

The online version contains supplementary material available at 10.1007/s00284-021-02394-z.

## Introduction

Adverse conditions due to biotic and abiotic stresses are the main factors limiting agricultural production and productivity [1–4]. Salinity is a major factor affecting soil fertility and limiting the growth and survival of plants in various parts of the world, particularly in arid and semi-arid areas where high evaporation and low precipitation make irrigation necessary to meet plant water needs [5]. About one-third of irrigated land is affected by salinity [4], mainly because of high temperatures, lack of fresh water and/or poor quality, salt-rich irrigation water [3] and poor management of irrigation [6]. Thus, salt accumulation in soils and groundwater has threatened productivity and soil sustainability [7] and the adverse effect of salinity on plant growth is well established [3]. Plant responses to NaCl stress include a series of changes at the molecular, biochemical and physiological levels, causing a disturbance of the homeostasis and the distribution of ions in the cell and denaturation of structural and functional proteins [4, 8]. Plants often face rapid fluctuations and adversity of environmental conditions due to their intrinsic metabolic abilities [9]. Plants also have the potential to reduce the impact of environmental stresses through the microbiome they harbour [4, 10]. The microbiota provides plants fundamental support for nutrient acquisition, disease resistance and abiotic stress tolerance [4, 11]. Its interaction with plants evokes various types of local and systemic responses that improve the metabolic capacity of plants to fight abiotic stresses [4, 10–12].

Legumes are a fundamental element of sustainable agriculture and can offer many economic and environmental benefits. They are very nutritious foods for people and essential nutrients for ecosystems [4]. The integration of legumes into agricultural techniques, such as intercropping, cover crops and crop rotation can restore soil health by their ability to fix nitrogen in a symbiotic interaction with the rhizobia of the soil. Based on their ability to grow on soils low in nitrogen, they can be used effectively to improve saline soil fertility and contribute to the reintroduction of agriculture on these lands [13]. In this line, *Sinorhizobium meliloti* (strain 1021) and its symbiotic interaction with *Medicago sativa* is a widely recognized model system for studies of symbiosis and nitrogen fixation. However, salt stress imposes a significant limitation of productivity in legumes. Salinity affects the infection process by inhibiting the growth of absorbent hairs, by decreasing the number of nodules per plant and the amount of nitrogen fixed per unit weight of nodules. Thus, in saline soils, legume yield is reduced due to unsuccessful symbiosis [14].

Plant growth-promoting rhizobacteria (PGPR) are a group of rhizosphere-colonizing bacteria that enhance plant growth, increase yield, improve soil fertility and reduce pathogens and biotic or abiotic stresses. PGPRs help plants by producing growth phytohormones, solubilizing phosphate and by providing antagonism to phytopathogenic microorganisms by producing siderophores, antibiotics and antifungal compounds [4, 10–12]. Actinobacteria are widely present in the rhizosphere of plants and produce various agro-active compounds. In the recent years, this group of bacteria, based on its high antimicrobial potential and its dominant saprophytic nature in the soil, has attracted much attention [15]. Actinobacteria promote plant growth by producing phytohormones (Indole Acetic Acid; IAA), siderophores and solubilizing phosphate. Some genera, such *Streptomyces* exert an immense biocontrol effect on various phytopathogens [14]. By these intrinsic abilities, they colonize successfully plants root systems by releasing many hydrolytic enzymes and can survive in hostile conditions by forming spores. Actinobacteria have long been considered as free-living soil inhabitants, but recently the importance of their complex interactions with plants and other organisms is being discovered and widely documented [16].

Actinobacteria have beneficial effects on nodulation and growth of legumes. Tokala et al*.* [17] found that the soil isolates *Streptomyces lydicus* WYEC108 increased the number of nodules, height and weight of pea (*Pisum sativum* L.) plants. Similarly, co-inoculation of soybean with *Streptomyces* sp. [18] or *Nocardia* sp. [19] and *Bradyrhizobium japonicum* improved the growth of soybean plants. Specific studies on alfalfa have shown growth enhancement associated with an application of *Micromonospora* spp. [20] alone or in co-inoculation with *Sinorhizobium meliloti* strain 1021. Solans et al. [21] found that actinobacteria other than *Frankia* isolated on the surface of the root nodules of *Discaria trinervis* improved the nodulation of alfalfa in the presence of high levels of nitrogen in the soil which usually inhibit nodulation. A comparative study of total bacterial profiles in soil with those specific for actinobacteria indicates an abundance of actinobacteria in the rhizosphere of legumes [22]. Subsequent studies have shown the beneficial effects of these isolates on legume growth and generated interest in their potential use as co-inoculants with rhizobia in legume crops [22].

Our primary results documented that multiple PGP traits of the salinity-tolerant actinobacteria, can increase alfalfa growth under saline conditions, in the presence or absence of symbiotic rhizobial bacteria. This finding highlights their possible use as biofertilizers for improvement of plant development, health and productivity in saline soils [23]. Therefore, this study aimed to enhance the colonization of the rhizosphere of *Medicago sativa* host plant by strains of *Sinorhizobium meliloti* under salt stress after co-inoculation with symbiotic and non-symbiotic actinobacteria isolated from different niches and having PGP activities. The Actinobacterial effect is checked through successful establishment of nitrogen-fixing symbiosis, particularly the formation of nodules and by the morphological and biochemical variations of the host plant.

## Materials and Methods

### Rhizobial Strains

Rhizobial strains *Sinorhizobium meliloti* 1021 (R1) and *S. meliloti* 2011(R2) have been obtained from Prof. Eric Boncompagni (University of Nice Sophia Antipolis, France) [24] and used in this study. Rhizobial strains have been cultured on mannitol yeast extract agar media and incubated for optimal growth at 28 ± 2 °C for 48 h. For storage, cultures have been stored at 4 °C on agar media until subsequent use (Table 1) [25].

**Table 1:** Origin and isolation details of actinobacterial and rhizobial strains used in the study

Bacterial group	Code	Isolation method	Type of samples	Number of selected isolates/total isolates	Geographical Location details
Actinobacteria group	MS1	ISP2 medium (28 ± 2 °C/from 2 to 15 days) [25]	Sand	4/20	Melghir Sebkha of Biskra region (34° 17′ 56.6″ N 6° 21′ 54.56″ E) Saline and arid area EC = 54.2 ± 2.7mS/cm, pH 7.56 ± 0.35
	MS2				
	MS3				
	MS4				
	Ag1	BAP medium (28 ± 2 °C/from 7 to 30 days) Re-streaked on ISP2 medium [25]	Nodules of *Alnus glutinosa*	1/13	Guerbes Senhadja of Skikda region (36° 55′ N 7° 16′ E) Humid area
Rhizobia group	R1 R2	YEM medium (28 ± 2 °C/2 days)	Symbiont of Alfalfa	2	–

### Sand Sampling

Five samples of sands were obtained from two geographically distinct locations. The sand of “Melghir Sebkha” located in South East Biskra region [34° 17′ 56.6″ N 6° 21′ 54.56″ E. pH 7.56 ± 0.35, electrical conductivity (EC) = 54.2 ± 2.7 mS/cm] representing saline and arid area and the nodules of *Alnus glutinosa* which grow in the humid area in front of Guerbes Senhadja river of Skikda region (36° 55′ N 7° 16′ E). No permission was required to collect such samples of sands.

### Isolation and Cultivation of Actinobacteria Strains

Two methods were used for bacterial isolation; firstly, the bacteria were isolated from the sand of “Melghir Sebkha”. The bacterial isolation was performed by homogenizing 10 g of sand in 90 mL of sterile physiological water and vigorous shaking for 1 h. The resulting supernatant was diluted in tenfold series (10^−1^ to 10^−5^) and plated out on two different media, International *Streptomyces* Program 2 ISP2 medium and Luedemann medium [25] supplemented with cycloheximide (50 µg/mL). Plates were then incubated at 28 ± 2 °C for 2 weeks. Growing colonies with different morphologies were re-streaked on new Petri dishes until obtaining pure bacterial cultures. Twenty colonies have been collected and tested for their PGP activities. Based on the PGP activities of the bacterial collection 4 strains designated MS1, MS2, MS3 and MS4 were selected for further characterization and plant inoculation studies (Table 1).

The second bacterial isolation was from the second sample, the nodules of *Alnus glutinosa* were harvested from plant roots after the removal of rhizosphere soil, washed in sterile distilled water, surface-sterilized by H_2_O_2_ (30% vol/vol) immersion for 10 min and rinsed with sterile distilled water. Each lobe of nodules was incubated in 5 mL of BAP medium and incubated at 28 ± 2 °C for several weeks with daily removal of culture tubes contaminated with fast-growing bacteria [25]. Thirteen bacterial cultures were obtained and re-streaked on ISP2 agar medium for further growth at 28 ± 2 °C for 2 weeks. All bacterial cultures were screened for their PGP activities and strain Ag-1 selected for subsequent characterization and plant inoculation experiments (Table 1). All isolates were maintained on plates for short-term storage and in medium supplemented with 30% glycerol at −80 °C for long-term storage.

### Identification of Actinobacteria by 16S-rRNA Gene Amplification, Sequencing and Phylogenetic Analysis

Bacterial genomic DNA was extracted by a CTAB–SDS lysis protocol [1]. PCR amplification of the 16S-rRNA gene was performed using the universal primers 27 F (5′-AGAGTTTGATCMTGGCTCAG-3′) and 1496R (5′-CTACGGCTACCTTGTTACGA-3′) [26]. PCR mixture, amplification conditions and PCR products sequencing were conducted according to Prospero et al. [2]. Partial 16S-rRNA sequences of the isolates were compared with available sequences in the National Centre for Biotechnology Information (NCBI) database (http://www.ncbi.nlm.nih.gov) by BLAST search. Actinobacterial 16S-rDNA sequence alignment and phylogenetic analysis were performed using standard procedures detailed in Mlaik et al. [26].

### Biochemical Characterization

Bacterial strains phenotypic screening was performed using different carbon sources and evaluated using standard methods. Degradation of sugars and amino acids used in the time course of the study was evaluated on minimal salt medium [10, 11]. To detect putative extracellular enzymes production of selected isolates, 10 µL of each bacterial suspension culture was plated in specific media. Amylase production was performed on starch media, protease production was performed on skim milk media, chitinase was checked on colloidal chitin agar and cellulase production was evaluated on CMC agar [10–12]. After incubation at 28 ± 2 °C for 7 days, positive isolates (after adding revealing reagent) were identified by the presence of a clear zone halo around bacterial colonies suggesting enzyme production by the bacterial isolate. Three replicates were performed per experiment.

### Stress Tolerance Studies

Several conditions were used to optimize the growth contention for the bacteria strains used in the current study. First, the ability of bacterial strains to tolerate salt stress, hydric stress and different pH values was performed on ISP2 broth for actinobacteria and YEM broth for rhizobia, supplemented with NaCl (0, 200, 400, 600, 800, 1000 and 1200 mM), PEG_8000_ (10, 20, 40 and 60%) and having different pH values (4, 7, 9 and 11), respectively*.* The media were inoculated with 100 μL of bacterial cultures and incubated at 28 ± 2 °C for 7 days. Bacterial growth was then determined by measuring the optical density at 600 nm using a spectrophotometer (Spectronic Genesys 20 Visible Spectrophotometer, Setif, Algeria) and plating on solid media with similar results.

The ability of the strains to tolerate different temperatures was performed by incubating the bacterial cultures at 4, 30, 37, 45 and 55 °C for 7 days and optical density measurement at 600 nm using a spectrophotometer (Spectronic Genesys 20, Setif, Algeria). Minimum of three replicates were performed per experiment.

### PGP Activities of Actinobacterial Strains

#### Growth on Nitrogen-Free Medium

Molecular nitrogen fixation was tested by the ability of the bacterial strains to grow on nitrogen-free medium, the DF salt minimal medium [12].

#### Ammonia (NH_3_) and Hydrogen Cyanide (HCN) Production

Ammonia production was revealed by the addition of Nessler’s reagent (0.5 mL) giving a yellow-to-brown colour of peptone water inoculated by bacterial cultures and incubated at 28 ± 2 °C for 7 days [12].

The production of hydrogen cyanide (HCN) was performed on nutrient agar supplemented with glycine (4.4 g/L) [12]. The medium was inoculated with the bacterial strains. Whatman paper impregnated with a solution of sodium picrate (5% picric acid and 2% sodium carbonate) was placed inside the lid of the plates and then sealed with parafilm and incubated at 28 ± 2 °C for 7 days. The development of an orange-brown colour on Whatman paper indicated hydrogen cyanide production.

#### Phosphate Solubilization

The phosphate solubilization ability was tested on Pikovskaya (PVK) medium containing tricalcium phosphate (Ca_3_HPO_4_) as the sole source of phosphate. A volume of 10 μL bacterial culture was spotted on the surface of the PVK agar as described by [11]. After incubation at 28 ± 2 °C for 7 days, the diameter of the clear halo around the colony was measured and used to estimate bacterial ability for phosphate solubilization.

#### Siderophores Production

Siderophore production was tested in Chrome Azurol S (CAS) medium according to [10]. The actinobacterial inoculated King B liquid medium was incubated at 28 ± 2 °C for 72 h and the resulting cultures were centrifuged at 5000 rpm for 30 min. 500 μL of the supernatant was then mixed with 500 μL CAS solution and OD measured at 630 nm after 20 min of incubation. The percentage of siderophores was evaluated using the following formula: S_t_-S_e_/S_t_ × 100, where S_t_ was the OD of the CAS solution (intense blue colour, control) and S_e_ was the OD of the test solution (light blue to orange colour depending on the intensity of production).

#### Indole Acetic Acid (IAA) Production

The production of indole acetic acid (IAA) was tested on Dworkin and Foster (DF) medium supplemented with 1 g/L tryptophan according to Slama et al. [10]. The cultures were then incubated at 28 ± 2 °C for 7 days and a colourimetric assay was performed using the method detailed in Slama et al. [10]. Briefly, the cultures were centrifuged at 5000 rpm for 20 min and 1 mL of supernatant was mixed with 2 mL Salkowski reagent (50 mL perchloric acid and 1 mL 35% FeCl_3_ 0.5 M). The OD was measured at 530 nm. Concentrations of IAA were determined using a calibration curve prepared from an IAA solution in the range 0 to 10^−5^ M.

### Effects of Bacterial Inoculation and Co-inoculation Experiments on the Nodulation of *Medicago sativa* L. Plants

#### Plant Materials

*Medicago sativa* L. seeds were obtained from the “Institut Technique des Grandes Cultures” (I.T.G.C.) of the city of Setif, Algeria. Alfalfa seeds were surface sterilized using successive incubations in ethanol 70% for 30 s, 4% sodium hypochlorite for 3 min followed by 4 successive washings by sterile distilled water [4]. Surface sterilized seeds were then germinated in dark, in Petri dishes amended with 0.8% water agar solution and incubated at 28 °C for 48 h.

#### Bacterial Inocula Preparation

Actinobacterial and rhizobial strains were grown on liquid ISP2 and YEM media as previously described and incubated at 28 ± 2 °C for 2 to 7 days. Bacterial cultures densities were then calibrated at 10^8^ cells/mL using a specific correlation between OD and bacterial count in the medium [4].

#### Bacterial Inoculations and Co-inoculations and Plant Growth Conditions

Surface sterilized alfalfa seeds were sown in sterilized internal surface plastic pots (by several washes using ethanol 70%). Pots were then filled with equal quantities of sand that was previously thoroughly washed with sterilized distilled water and autoclaved (121 °C for 1 h during three cycles separated by 24 h). Surface sterilized seeds were sown aseptically at 1 cm depth at a density of three seeds per pot. After 1 week, alfalfa plants were inoculated with 1 mL of each isolate bacterial suspension taken during the exponential growth phase and calibrated at 10^8^ cells/mL [4]. Inoculation and co-inoculations were performed using rhizobial strains R1 and R2 and actinobacterial strains MS1-4 and Ag1 alone or by joint inoculation with one rhizobial and each actinobacterial selected strains (Table 2). Pots were then placed in a growth chamber with 16 h day/8 h night photoperiod and 26/18 °C day/night temperature. Pots were watered twice weekly with a nitrogen-depleted nutritive solution. The experiment had been repeated six times and after 2 months of culture alfalfa plantlets were collected and the presence of nodules recorded.

**Table 2:** Different treatments used in the experiment

Treatment	Bacterial strains
C−	Non-inoculation (negative control)
C+	Non-inoculation with addition of KNO3 (0.5 M) (Positive control)
R1	*Sinorhizobium meliloti*
R1+MS	R1+MS1, R1+MS2, R1+Ag1, R1+MS3, R1+MS4
R2	*Sinorhizobium meliloti*
R2+MS	R2+MS1, R2+MS2, R2+Ag1, R2+MS3, R2+MS4

#### Bacterial Inoculations and Co-inoculations and Plant Growth Conditions Under Salt Stress

Based on the results obtained in the “Bacterial inoculations and co-inoculations and plant growth conditions” section experiments, three actinobacterial strains MS1-3 having induced nodulation had been selected to evaluate their efficiency in co-inoculation experiments with rhizobial strains under salt stress. Experiments had been conducted as previously described in the section “Bacterial inoculations and co-inoculations and plant growth conditions” except that plants had been grown with and without salt stress. The experimental set-up was explained in Table 3. Briefly, after 1 week of alfalfa plant growth, bacterial inoculation and co-inoculations had been performed using 1 mL of bacterial suspensions of each isolate at a density of 10^8^ cells/mL. Inoculations and co-inoculations had been repeated three times during 3 weeks and the pots were watered two times a week with a nitrogen depleted nutritive solution. Two weeks post-inoculation and co-inoculation pots of the second group (100 mM NaCl) were watered with the 100 mM saline solution once a week during 1 month (4 times).

**Table 3:** Different treatments used in NaCl stress experiment

Treatment without NaCl	Treatment with 100 mM NaCl	Bacterial strains
C−	C−	Non-inoculation (negative control)
C+	C+	Non-inoculation with addition of KNO_3_ (0.5 M) (positive control)
R1	R1	*Sinorhizobium meliloti* (R1)
R1+MS	R1+MS	R1+MS1, R1+MS2, R1+Ag1
R2	R2	*Sinorhizobium meliloti* (R2)
R2+MS	R2+MS	R2+MS1, R2+MS2, R2+Ag1
MS	MS	MS1, MS2, Ag1

#### Monitoring of Morphological Parameters

After 2 months of culture, alfalfa plantlets were collected and their growth evaluated by estimation of root and shoot fresh weight (g), plantlet aerial part height (cm), root length (cm), leaves and nodules numbers.

#### Estimation of Photosynthetic Chlorophyll Content

Photosynthetic chlorophyll contents have been determined using the procedure described by Rekik et al. [3]. Briefly, 0.5 g of leaf material of each sample were cut in 1 mm^2^ pieces and homogenised in 10 mL 80% acetone and incubated at −10 °C overnight. Extract was then centrifuged at 14,000 rpm for 5 min and optical density of the supernatant estimated at 663, 645 and 470 mM. Chlorophyll a (Ch_a_), b (Ch_b_) and total chlorophyll a and b contents (Ch_a+b_) and carotenoids expressed in mg/g were evaluated according to the following equations:

$${\text{Ch}}_{{\text{a}}} \left( {{\text{mg}}/{\text{g}}} \right) \, = { 12}.{\text{41 OD}}_{{({663})}} {-}{ 2}.{\text{59 OD}}_{{({645})}}$$

$${\text{Ch}}_{{\text{b}}} \left( {{\text{mg}}/{\text{g}}} \right) \, = { 22}.{\text{9 OD}}_{{({645})}} {-}{ 4}.{\text{68 OD}}_{{({663})}}$$

$${\text{Ch}}_{{{\text{a}} + {\text{b}}}} = {\text{ CH}}_{{\text{a}}} + {\text{ CH}}_{{\text{b}}}$$

$${\text{Carotenoids }}\left( {{\text{mg}}/{\text{g}}} \right) \, = \, \left[ {\left( {{1}000 \, \times {\text{ OD}}_{{({47}0)}} } \right) \, {-}{ 1}.{\text{82 Ch}}_{{\text{a}}} {-}{ 85}.0{\text{2 CH}}_{{\text{b}}} } \right)]/{198}$$

#### Soluble Amino Acids and Proline Content

Amino acids and proline extraction procedures have been detailed in Cherif-Silini et al. [4]. Briefly, 0.5 g plantlet fresh material was harvested in 5 mL methanol:chloroform:water solution 60:25:15 respectively. The samples were then heated in a water bath at 60 °C for 2 h and centrifuged at 10,000 rpm for 10 min. The supernatant was then used to estimate soluble amino acids and proline contents.

For soluble amino acids content, 1 mL of supernatant was added to 1 mL of acetate buffer (pH 4.3) and 1 mL of ninhydrin solution (5% in ethanol). Samples were then vigorously shaken and heated in a water bath at 95 °C for 15 min. OD was then measured at 570 nm and concentration of soluble amino acids obtained using glycine as standard.

Free proline content had been measured according to Cherif-Silini et al. [4]. Briefly, 1 mL of the supernatant described earlier had been added to 4 mL of ninhydrin solution (5% in ethanol), 4 mL of glacial acetic acid and 1 mL of sterile distilled water. The mixture was then heated in a water bath at 90 °C for 45 min and left to settle and reach room temperature. Then, 4 mL toluene had been added to the mixture and OD of the organic phase estimated at 520 nm. Free proline concentration was determined using a calibration curve with known amounts of free proline added.

### Statistical Analysis

All the experiments were performed in triplicate, the results expressed as means ± standard deviation (SD) The statistical analysis of the data was conducted using GraphPad prism 8, one-way- and two-way ANOVA was used to identify the variance between different treatments. The results were considered significant when *P* values were less than 0.05. post hoc Tukey’s HSD test comparison tests were conducted when a significant difference was encountered.

#### GenBank Accession Numbers

GenBank accession numbers of the bacteria isolates MS-1, MS-2, Ag-1, MS-3 and MS-4 are MK894856, MK894855, MN420819, MN005930 and MK894857, respectively.

## Results

### Isolation, Morphological and Molecular Characterization of Rhizobial and Actinobacterial Strains

**Fig. 1:**
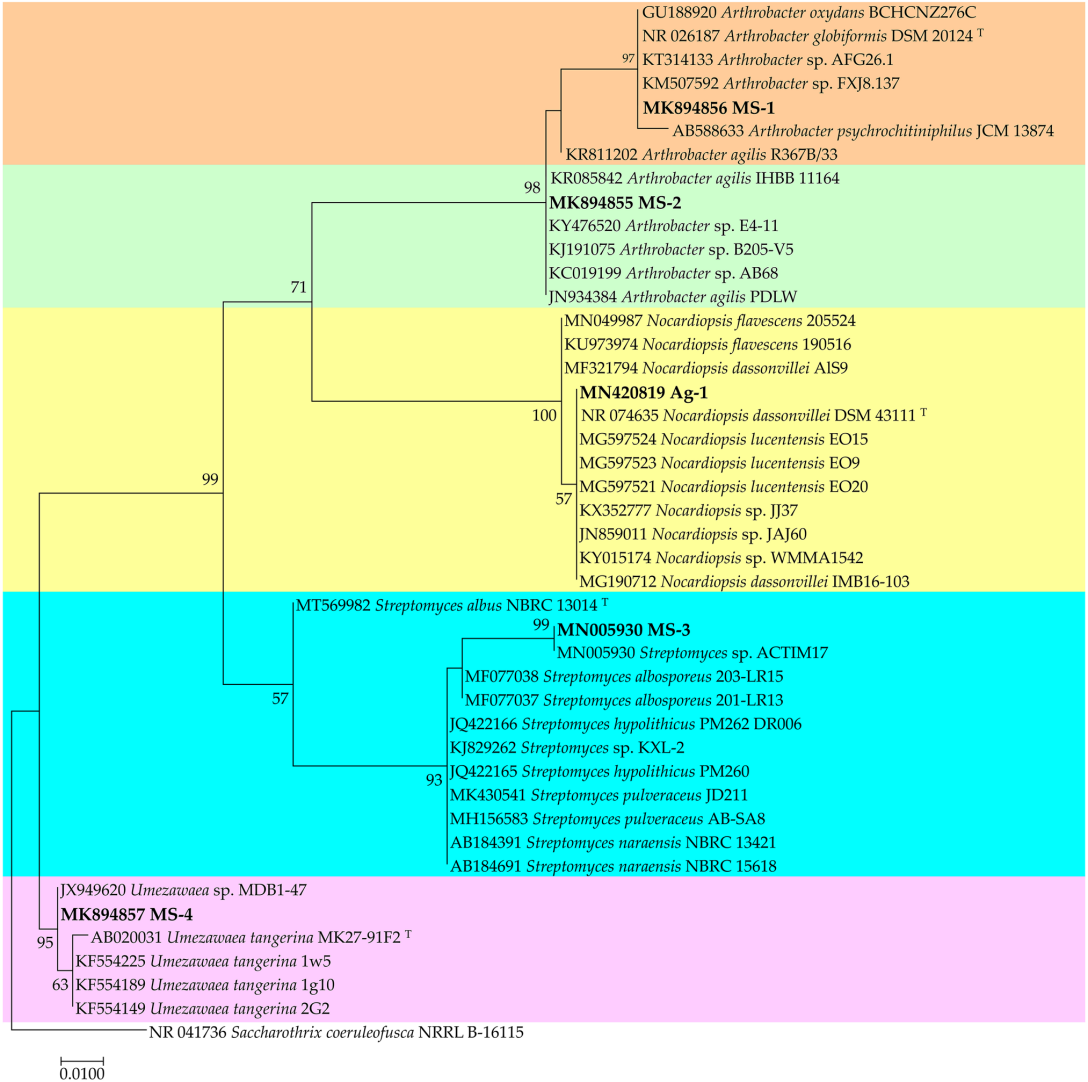
Maximum likelihood phylogenetic tree of actinobacterial strains (Ag1, MS1, MS2, MS3 and MS4). The tree was rooted to *Saccharothrix coeruleofusca* NRRL B-16115. Bar, the number of expected changes per site. *T* refers to type strains

Growth of rhizobial strains *S. meliloti* R1 and R2 on yeast mannitol agar (YEM) at 28 °C for 48 h revealed small colonies (1 mm in diameter) of rod-shaped cells. Actinobacterial strains selected cultured on ISP2 media at 28 °C after 2 to 7 days of culture showed smooth colonies having an orange- and red-coloured non-diffusible pigments for MS1 and MS-2, respectively. However, colonies of Ag1, MS3 and MS4 had rough aspects and presented aerial mycelia and a white or grey coloured substrate mycelium. The morphological microscopy of these strains was additionally filamentous (Fig. S1). Majority of the rhizobial and actinobacterial strains catabolized numerous carbon sources and produced different enzymes, such as amylase, protease, chitinase and cellulose (Table S1). 16S-rDNA phylogenetic analysis unambiguously documented that MS1 and MS2, MS3 and MS4 had a homology with the genera *Arthrobacter*, *Streptomyces* and *Umezawaea*, respectively, while Ag-1 lied within the genus *Nocardiopsis* see (Fig. 1).

### Physiological Characterization of Rhizobial and Actinobacterial Strains

**Fig. 2:**
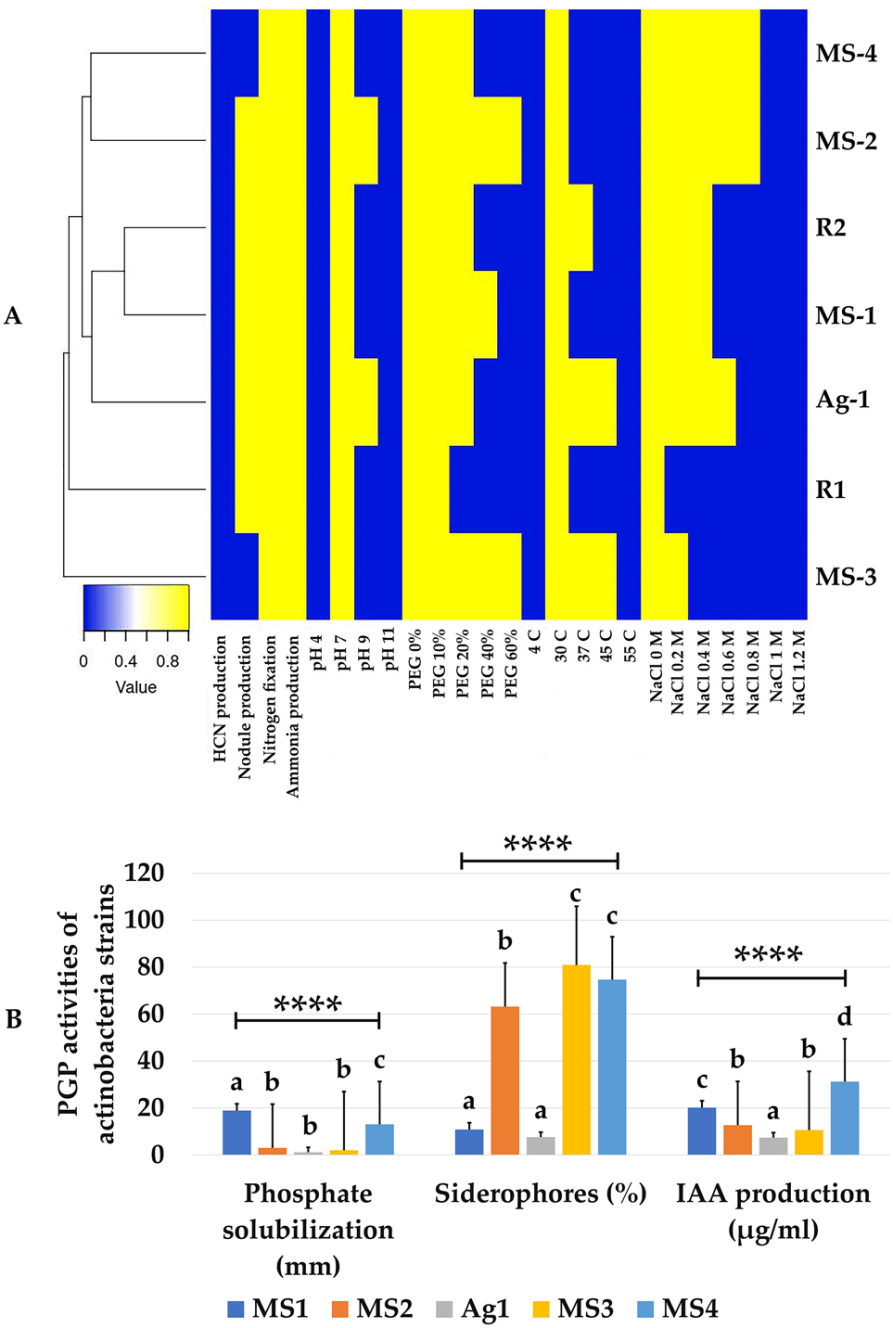
**a** PGP activities and stress tolerance of bacterial and actinobacterial strains used in the study. **b** PGP activities of actinobacterial strains. The data present mean ± standard error. Bars labelled with different letters are significantly different among the treatments at *P*  < 0.05 using Tukey’s HSD test

In this study, we had optimised the condition for the bacterial strains, the effect of initial pH and temperature were studied to identify the optimum condition for these bacteria. All strains had been characterized at the physiological level and results were presented in Fig. 2a. Actinobacteria strains were able to grow at pH 7, and can tolerate the extreme pH values. Except MS2 and MS3 were able to tolerate pH values of 9 (Fig. 2a). Among all strains tested for tolerance to different concentrations of PEG rhizobial strains were sensitive to hydric stress. *S. meliloti* R1 could not grow beyond a 10% PEG concentration compared to *S. meliloti* R2 strain that could tolerate up to 20% PEG. Actinobacteria on the other hand seemed more adapted to hydric stress mirrored by their ability to grow at high PEG concentrations. MS2 and MS3 for example tolerated up to 60% PEG concentrations (Fig. 2a). All examined rhizobacterial and actinobacterial strains were sensitive to extreme temperatures (4 and 55 °C). Optimal growth temperature for all actinobacterial strains was around 30 °C with strains Ag1 and MS3 was able to grow up to 45 °C (Fig. 2a). Growth of the strains at salt concentrations ranging from 0 to 1200 mM revealed a good ability of MS2 and MS4 to tolerate up to 800 mM salt in the culture medium. NaCl sensitivity was noticed for *S. meliloti* R1 that did not tolerate salt at any of the salt tested concentrations. *S. meliloti* R2 and MS1, however, were able to grow up to 400 mM salt concentrations. MS3 was also able to tolerate up to 200 mM salt (Fig. 2a).

### PGP Activities of Rhizobial and Actinobacterial Strains

All selected actinobacterial strains produced ammonia and efficiently fixed nitrogen, however, none of them was able to produce HCN (Fig. 2a). Siderophore, IAA and phosphate solubilization were detected in all actinobacterial strains but to varying levels. IAA production varied from 7.3 to 31.2 µg/mL with strains MS1 and MS4 being the best producers with 20.15 and 31.18 µg/mL, respectively. Siderophore production reached high levels of activity mainly for strains MS2, MS3 and MS4 producing 63.13, 80.9 and 74.62%, respectively (Fig. 2b). Phosphate solubilization was observed only for two strains MS1 and MS4 with halo diameters reaching 18 and 13 mm, respectively. All other strains had weak phosphate solubilization abilities (Fig. 2b).

### Morphological Parameters of Bacterial Plants Inoculation and Co-inoculation Experiments

**Fig. 3:**
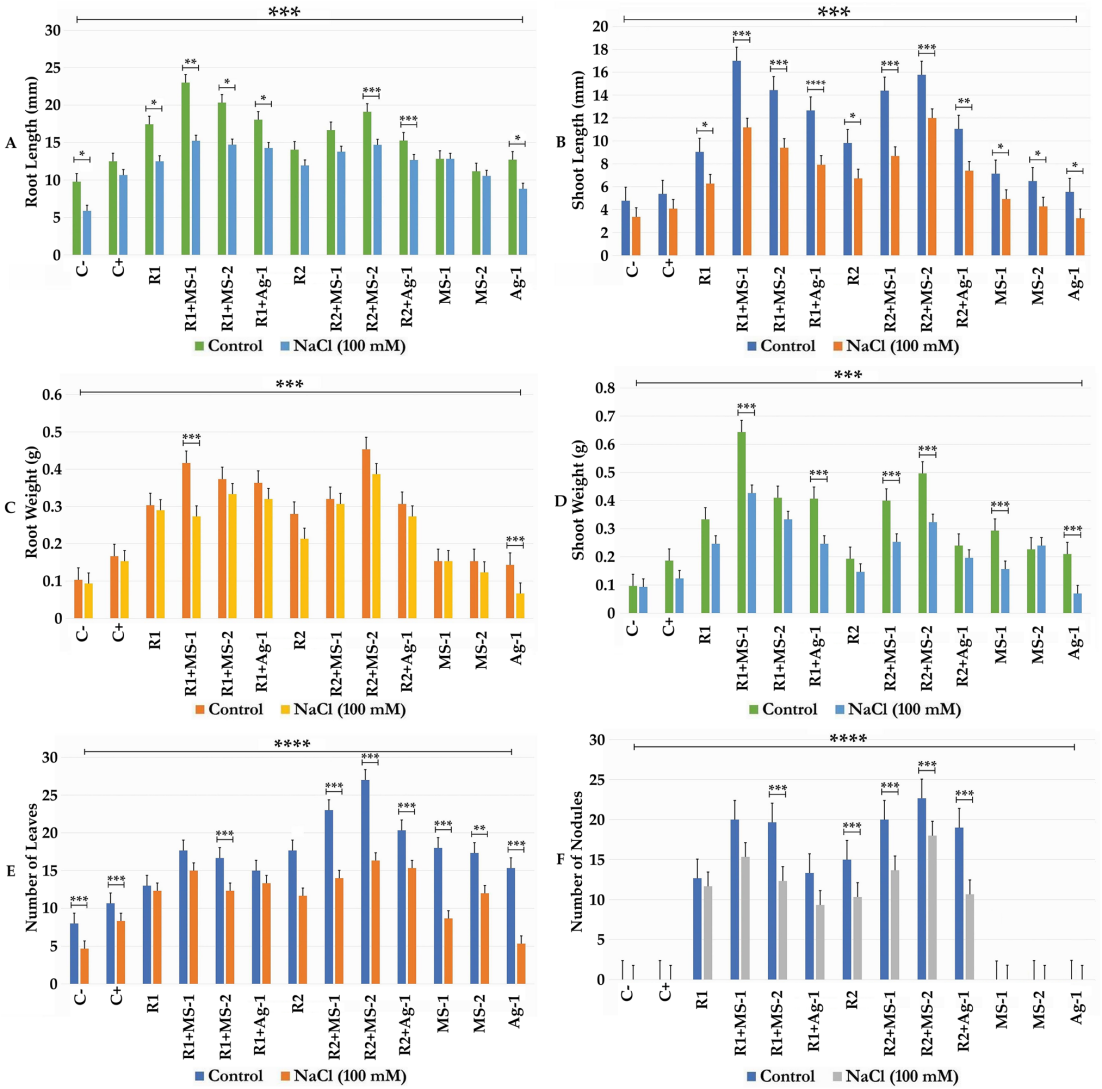
**a**, **b** Root length and shoot length (mm) of *Medicago sativa* against different treatments of rhizobacterial and actinobacterial strains and NaCl treatments. **c**, **d** Root weight and shoot weight (g) of *Medicago sativa* against different treatments of rhizobacterial and actinobacterial strains and NaCl treatments. **e, f** Number of leaves and nodules of *Medicago sativa* against different treatments of rhizobacterial and actinobacterial strains and NaCl treatments. The data present mean ± standard error. Bars labelled with asterisk are significantly different among the treatments at *P*  < 0.05 using ANOVA analysis

Variance analysis of root and shoot length, root and shoot fresh weight and leaves and nodule numbers documented that bacterial inoculation was beneficial to alfalfa plants and that salinity severely and significantly interfered with these growth parameters (*P* < 0.05). In both inoculation and co-inoculation experiments with and without salt stress, morphological parameters of alfalfa plants inoculated with rhizobia alone or co-inoculated with actinobacteria showed a significant effect of co-inoculation of *S. meliloti* R1 and R2 strains with actinobacterial isolates MS1, MS2 and Ag1 (Fig. 3). Bacterial inoculation experiments and plant growth under salt stress results indicated that salinity affected the shoot part and the root length. However, the treatment with co-inoculation showed a significant increase *P* < 0.0001 in the shoot (131–256%) and root length (56–135%) in comparison to the negative control. The optimum length was achieved in the inoculant by the plants *S. meliloti* R1 and R2 strains with actinobacterial isolates MS1, MS2 and Ag1 (Fig. 3a, b). Plant aerial parts and roots fresh weight were significantly improved in alfalfa plants co-inoculated with *S. meliloti* R1 and R2 strains. The actinobacterial strains MS1, MS2 and Ag1 showed a significant increase of 2 to 3 times the weight of shoots and roots in the absence and in the presence of salt stress as compared to the negative control (Fig. 3c, d). In addition, bacterial co-inoculation experiments improved the numbers of leaves and nodules. The plants co-inoculated showed a number of nodules increased by 61 and 51% for the strains R1 and R2, respectively as compared to the plants inoculated alone (Fig. 3e,f). In experimental conditions with NaCl stress, nodule number was significantly higher with R1 (36%) and R2 (80%) than control inoculations under NaCl stress (Fig. 3f). Nodulation ability of actinobacterial strains in co-inoculation experiments with *S. meliloti* R1 and R2, clearly documented that three strains MS1, MS2 and Ag1 were able to allow nodule formations on *Medicago sativa* roots (Fig. 3f).

### Biochemical Parameters of Bacterial Plants Inoculation and Co-inoculation Experiments

**Fig. 4:**
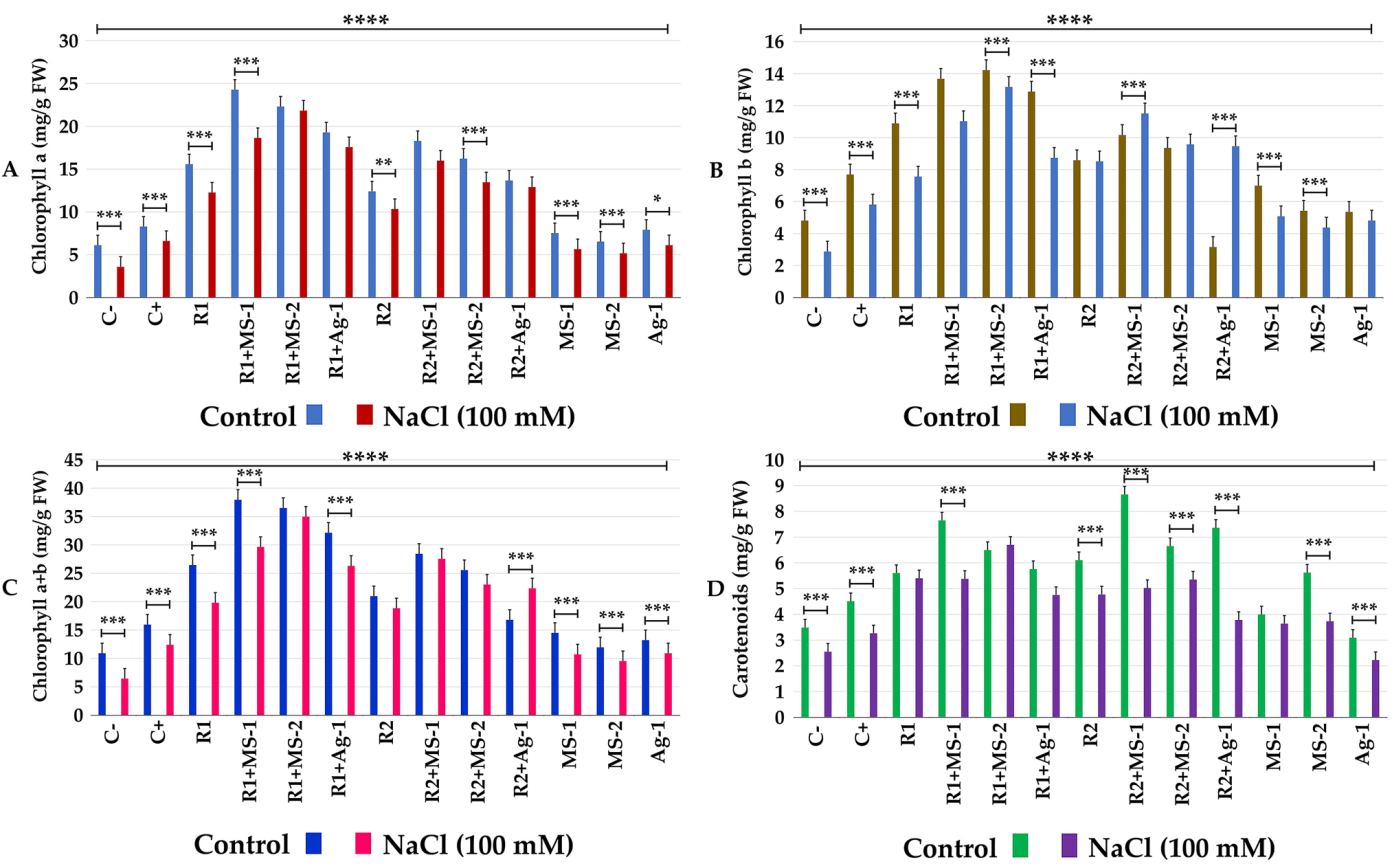
**a** Chlorophyll a, **b** Chlorophyll b, **c** Chlorophyll a + b, **d** Carotenoids (mg/g FW) of *Medicago sativa* against different treatments of rhizobacterial and actinobacterial strains and NaCl treatments. The data present mean ± standard error. Bars labelled with different letters are significantly different among the treatments at *P*  < 0.05 using Tukey’s HSD test

**Fig. 5:**
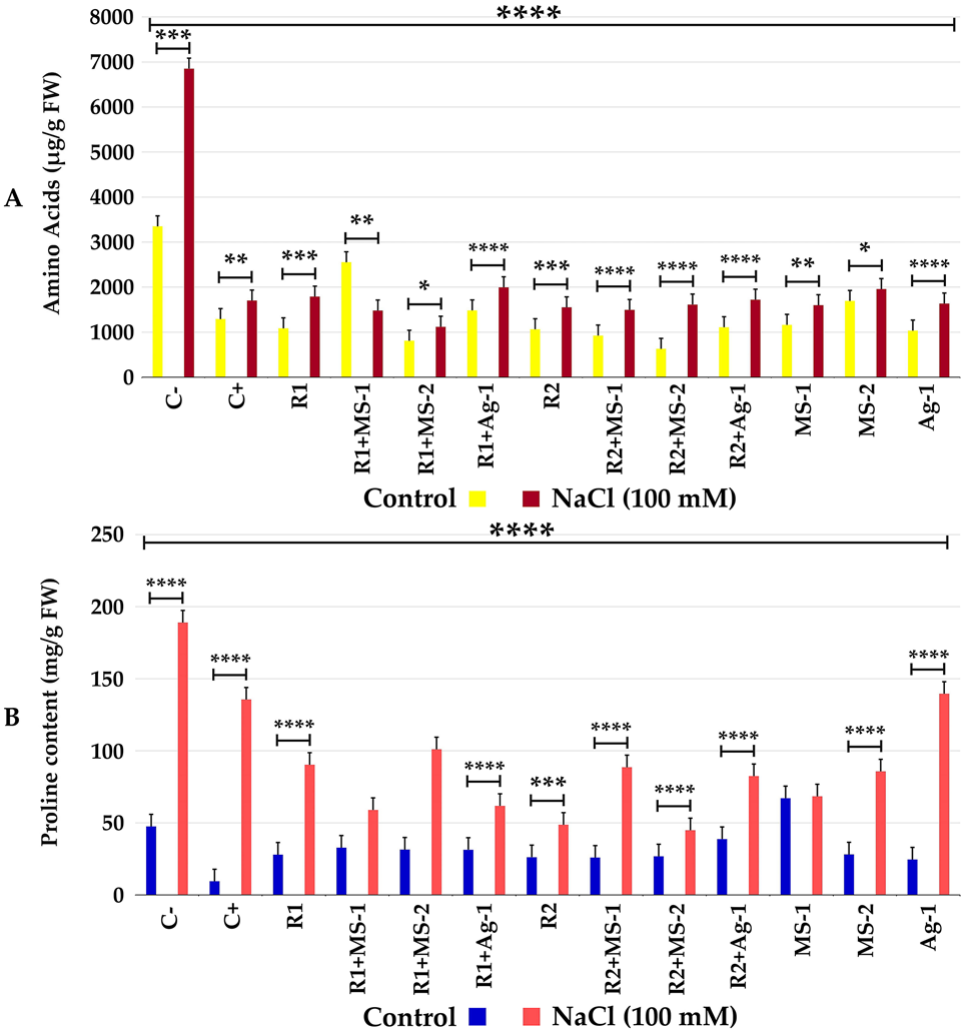
**a** Amino acids and **b** proline content (µg/g FW) of *Medicago sativa* against different treatments of rhizobacterial and actinobacterial strains and NaCl treatments. The data present mean ± standard error. Bars labelled with different letters are significantly different among the treatments at *P*  < 0.05 using Tukey’s HSD test

Chlorophyll a, b (Fig. 4a, b) and total chlorophyll (Fig. 4c) and carotenoid contents (Fig. 4d) were significantly improved in co-inoculated plants with and without NaCl stress. Proline accumulation and free amino acid levels in leaves of alfalfa plants co-inoculated with rhizobial and actinobacterial strains were significantly lower than control levels. These finding clearly illustrated that actinobacterial strains could mitigate salt stress that was reflected by lower levels of proline and free amino acids in leaves of inoculated and co-inoculated plants (Fig. 5a, b).

## Discussion

Biological nitrogen fixation plays a crucial role in improving soil productivity [27]. Therefore, nitrogen deficiency severely limits plant growth and productivity [5]. Rhizobium Legume Symbiosis is an economically sound nitrogen source providing necessary nitrogen amounts for efficient plant growth and development and an efficient sustainable agricultural practice as compared to external fertilizer supplies that are costly for the farmers and damaging to the environment [28, 29]. Unfortunately, numerous environmental factors limit nitrogen fixation affecting survival and rhizobia and the rate of infectivity [30, 31]. Recent trends in the field aiming the development of stress-tolerant crops suggest the use of plant growth-promoting (PGP) bacteria as an efficient mean to improve biological nitrogen fixation [32].

In the current study, three selected bacterial strains affiliated to the Actinobacteria phylum were able to enhance nitrogen-fixing symbiosis between *S. meliloti* and the legume *M. sativa*. The promotion of nitrogen-fixing symbiosis by actinobacteria has been widely documented [33]. Recent studies focus more on PGP potential of actinobacteria as compared to other bacteria, due to their relative abundance in the soil and their ability to produce a variety of metabolites. The selected strains showed these abilities, they can degrade various carbonaceous substances. These characteristics allowed not only to study their metabolic profile but also to understand their ability to compete and survive [34].

Also, it has been reported that a large part of their genome (~5–10%) is devoted to the production of secondary metabolites which help them to grow even in extreme conditions. Actinobacteria are widespread in aquatic and terrestrial habitats, including extreme habitats and hyper-arid desert soils [13]. This was clearly shown by the tolerance of these strains to abiotic stress, particularly aridity and salinity. Given the origin of their isolation, some strains can tolerate salt concentrations up to 800 mM, an alkaline pH 9, a temperature of 45 °C and a PEG osmotic pressure of 60%. Soil characteristics play a prominent role in the microbial selection process. Bacteria isolated from extreme environments can survive inhibitory parameters compared to those isolated from non-stressful habitats [35].

It is well documented that Actinobacteria, like other PGPRs, employ direct and indirect mechanics to boost crops growth and protection against diseases. In the present study, all our isolates produced IAA at variable rates. It is well documented that rhizospheric and endophytic actinobacteria can provide phytohormones to their plant hosts [16, 36]. IAA is the main phytohormone that boosts plant growth and development. IAA, besides being critical for nodule formation, have also been reported as essential trigger of cell proliferation and differentiation and formation. of vascular bundles [37]. As previously reported in previous studies, the phosphate solubilization ability was also present in our isolates [38]. It is worth noticing that in the report of Fernandez et al. [39] phosphate solubilizing bacteria have favourable effects on soybean growth. This improved the nutrient supply of *Rhizobium* symbionts and subsequent nodules formation. Other PGP traits have been shown in the selected actinobacteria like siderophores production. The genus *Streptomyces* was well known for its characteristic siderophores types, such as desferrioxamine and coelichelin [40] that not only play a major role in plant nutrition, but also provided plant protection via phytopathogen control. The ability to fix nitrogen in these strains, revealed by strains growth on nitrogen-free medium, has also been reported [41]. It has been documented that PGP actinobacteria can minimize and cope with the adverse effects of biotic and abiotic stresses [14, 42]. Numerous studies suggested that biotic abiotic stresses mitigation in plants by PGP actinobacteria is done through cell wall degrading enzymes (protease, cellulase, chitinase), secondary metabolite production, low molecular weight inhibitors substances (ammonia for example) and nutrients competition. Jog et al. [42] suggested that these modes of actions should be major properties of any effective fertilizer. In the present study, all selected strains showed multiple PGP traits, independently of their isolation origin. Except the Ag1 strain, all other strains were non-rhizospheric or endophytic bacteria and to our knowledge, this is the first study showing that this kind of bacteria can be used as a plant growth promoter.

Our results clearly showed the importance of actinobacterial inoculants in stimulating the growth of *M. sativa* plants. Improvement in nodulation and nitrogen fixation in alfalfa cultivars was related to the co-inoculation treatment. It was noted that plants co-inoculated with actinobacteria and *Sinorhizobium* had increased nodulation and plant growth compared to plants with single inoculation. Similar results have been previously reported by Gregor et al*.* [43] using *Streptomyces kanamyceticus* and *Bradyrhizobium japonicum* combination for inoculation of soybean. The authors reported an increased nodulation (55%) and nitrogen composition (41%) of soybean stems. In another study Soe and Yamakawa [44] reported an improved nodulation, nitrogen fixation and seed yield of different soybean varieties after co-inoculation of *Bradyrhizobium yuanmingense* MAS34 and *Streptomyces griseoflavus* P4. Moreover, the study of Volpin et al. [45] suggested the evidence of a more direct effect of *Azospirillum brasilense* on nodulation mediated by increased flavonoids exudation in the rhizosphere of alfalfa and common bean. Flavonoids are believed to be plant signalling molecules essential for the establishment of rhizobia–legume symbiosis. It is widely believed that legume nodules are often occupied by a phylogenetically diverse bacterial microbiome. These bacteria have wide effects on plant growth and health as well as nodule formation and nitrogen fixation. However, their precise ecological roles remain unknown [46]. A non-passive role of the non-rhizobial bacteria in nodules has been suggested including manipulation of the plant host [47]. Liu et al. [20] proved that some non-rhizobial bacteria improve the nodulation and nitrogen fixation of leguminous symbionts–rhizobia and help *Rhizobium* extending its host range. They selectively control the entry of bacteria in this specific niche, the root nodule and allow their survival [48]. Molecular studies have shown that many actinobacteria can occur as endophytes in various leguminous plants [11]. *Streptomyces* inoculants application to enhance plant nodulation and increase soil nitrogen content indicated their potent effect on rhizobia–legume symbiosis non-specifically [26]. However, in our case, *Streptomyces* did not seem to contribute to nodulation as compared to the genus *Arthrobacter* which not only improved nodulation, but also all the plant growth parameters, in the presence of *S. meliloti* R1 or R2.

Variance analysis showed that alfalfa growth parameters were negatively affected by salinity. Bacterial inoculation has been reported to reduce the undesirable effects of stress on plant growth [4]. Our results were in agreement with earlier findings that inoculated plants grew better and had higher biomass than non-inoculated plants under salt stress conditions. Legume–rhizobia symbiosis and the process of initiation of nodules on legumes were both sensitive to salt stress [49]. Salt stress inhibited the early stages of rhizobia–legume symbiosis. The adverse effects of salinity on symbiosis may also result from the suppression of nodule function and the reduction of plants’ ability to grow [49]. The increased growth of plants co-inoculated with non-rhizobial strains as compared to plants inoculated with the rhizobial strain *S. meliloti* alone can be attributed to the higher tolerance of actinobacteria strains to salinity and drought and to their greater ability to produce IAA hormone (MS1 and MS2).

Saline stress had also deleterious effect on biochemical parameters resulting in a decrease in chlorophyll concentration [50] due to the weakening of protein–lipid complexes [50] and consequently to the reduction in interception of light, an increase in the activity of chlorophyllase and the synthesis of nitrogen compounds that consumes a large quantity of nitrogen [51]. It was also known that higher concentrations of ions in saline soils accumulate in plant cells, inactivate enzymes and inhibit protein synthesis and photosynthesis [3].

Analysis of the leaf proline content revealed that salinity stimulated its intracellular synthesis. This synthesis was at the maximum level at 100 mM NaCl. Inoculation induced a decrease in leaf content under the effect of salt stress. Proline is the amino acid most widely accumulated by plants in saline soil [4]. Inoculated plants can improve their growth rate and salt tolerance and suppress the harmful effects of stress, allowing them to adapt to salt conditions [4]. The results of this study indicate that drought- and salinity-tolerant actinobacteria with multiple PGP traits can potentially increase alfalfa growth under saline conditions, in the presence or absence of symbiotic rhizobial bacteria. This knowledge will be useful to define strategies for the application of these bacteria as bio-inoculants, alone or associated with rhizobial bacteria. Such approaches will improve the performance or persistence of rhizobia and reduce the use of chemical fertilizers [28]. According to the results of this study, MS1 and MS2 bacteria can be suitable biofertilizers in the formulation of agricultural products improving plant development, health and productivity in saline soils, a necessary alternative for modern agriculture and sustainable development.

## Conclusions

Our study highlights the potential use of Actinobacteria and mainly those discovered in this study as biofertilizers in the formulation of agricultural products improving plant development, health and productivity in saline soils, a necessary alternative for modern agriculture and sustainable development.

## Supplementary Information

Below is the link to the electronic supplementary material.

Electronic supplementary material 1 (DOCX 253 kb)
