# Supplementary material for: Improvement of Medicago sativa Crops Productivity by the Co-inoculation of Sinorhizobium meliloti–Actinobacteria Under Salt Stress
Source: Curr Microbiol. 2021 Mar 1;78(4):1344–57. doi: 10.1007/s00284-021-02394-z (PMC7997840; doi:10.1007/s00284-021-02394-z)

Improvement of *Medicago sativa* crops productivity by the co-inoculation of *Sinorhizobium meliloti*-Actinobacteria under salt stress

Journal of Current Microbiology

Samira Saidi ^1^, Hafsa Cherif-Silini ^1^, Ali Chenari Bouket ^2^ Allaoua Silini ^1^, Manal Eshelli^3^, Lenka Luptakova^4^, Faizah N. Alenezi^5^, Lassaad Belbahri ^6,7,*^

1. Laboratory of Applied Microbiology, Department of Microbiology, Faculty of Natural and Life Sciences, University Ferhat Abbas Setif, Setif, Algeria

^2^ Plant Protection Research Department, East Azarbaijan Agricultural and Natural Resources Research and Education Center, AREEO, Tabriz, Iran

^3^ Food Science and Technology Department, Faculty of Agriculture, University of Tripoli, Tripoli, Libya

^4^ Department of Biology and Genetics, Institute of Biology, Zoology and Radiobiology, University of Veterinary Medicine and Pharmacy, Kosice, Slovakia

^5^ Department of Environmental Technology Management, College of Life Sciences, Kuwait University, Safat, Kuwait

^6^ NextBiotech, 98 Rue Ali Belhouane, Agareb, Tunisia

^7^ Laboratory of Soil Biology, University of Neuchatel, Neuchatel, Switzerland

^*^Corresponding Author: [lassaad.belbahri@unine.ch](mailto:lassaad.belbahri@unine.ch)

**Supplementary Table 1** Biochemical characterization of actinobacterial and rhizobial strains used in the study.

|  | MS1 | MS2 | Ag1 | MS3 | MS4 | R1 | R2 |
| --- | --- | --- | --- | --- | --- | --- | --- |
| Colony diameter (mm) | 3 | 4 | 5 | 3 | 4 | 1 | 1 |
| Color of colony or aerial mycelium | Orange | Red | Grey | White | Grey | White | White |
| Color of substrate mycelium | - | - | White | Beige | White | - | - |
| Microscopic morphology | Rod-coccus | Rod-coccus | Filamentous | Filamentous | Filamentous | Rod-coccus | Rod-coccus |
| Gram-reaction test | + | + | + | + | + | - | - |
| Diffusible pigments | - | - | - | - | - | - | - |
| Adonitol | - | - | + | - | - | + | - |
| Amygdaline | + | - | + | + | - | - | - |
| Arabinose | - | - | - | - | - | - | - |
| Cellobiose | - | + | - | - | - | - | - |
| Fructose | + | + | - | + | + | + | + |
| Glucose | + | + | + | + | + | + | + |
| Inositol | + | - | - | - | - | - | - |
| maltose | + | + | - | - | - | + | + |
| Mannose | + | + | + | + | - | + | + |
| Melibiose | - | - | - | + | - | - | - |
| Raffinose | - | - | + | - | - | + | + |
| Rhamnose | + | + | - | - | - | - | - |
| Sorbitol | - | - | - | + | - | - | - |
| Sorbose | - | - | + | - | - | - | - |
| Sucrose | + | - | - | + | - | + | + |
| Xylose | - | - | - | + | - | + | - |

**Supplementary Table 1** Continued.

|  | MS1 | MS2 | Ag1 | MS3 | MS4 | R1 | R2 |
| --- | --- | --- | --- | --- | --- | --- | --- |
| Citrate | + | + | + | + | + | + | + |
| β-galactosidase | + | - | + | + | + | - | - |
| Lysine decarboxylase | + | + | + | + | + | + | + |
| Ornithine decarboxylase | + | + | + | + | + | + | + |
| Arginine dihydrolase | + | + | + | + | + | + | + |
| Butanediol | + | + | - | + | + | - | - |
| Urease | + | - | + | + | + | + | + |
| Amylase | + | + | + | + | + | + | + |
| Protease | + | + | + | + | + | + | + |
| Chitinase | + | + | + | + | + | + | + |
| Cellulase | + | + | + | + | + | + | + |
| Gelatinase | + | + | - | + | + | + | - |

**Supplementary Fig. 1** Microscopy (upper part) and colony morphology (lower part) images of the actinobacterial strains MS1, MS2, Ag1, MS3 and MS4.


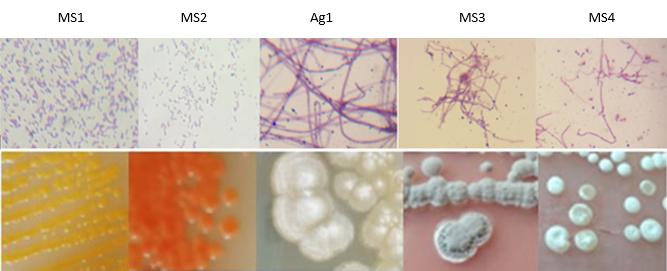

Supplement: Supplementary file 1 — Electronic supplementary material 1 (DOCX 253 kb) [file 284_2021_2394_MOESM1_ESM.docx]
